# Supplementary material for: Identification of photoperiod-induced specific miRNAs in the adrenal glands of Sunite sheep (Ovis aries)
Source: Front Vet Sci. 2022 Jul 22;9:888207. doi: 10.3389/fvets.2022.888207 (PMC9354845; doi:10.3389/fvets.2022.888207)
Supplement: Supplementary file 1 [file Data_Sheet_1.ZIP › Supplementary file/miRDeep2 file.zip!/miRDeep2 file/Know_Structure/SPLP42A1/oar-mir-181a-2.pdf]

miRBase precursor : oar-mir-181a-2
Total read count : 16031
oar-miR-181a read count : 15917
remaining reads : 114

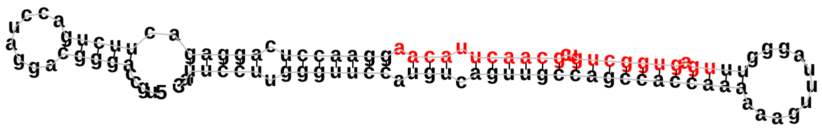

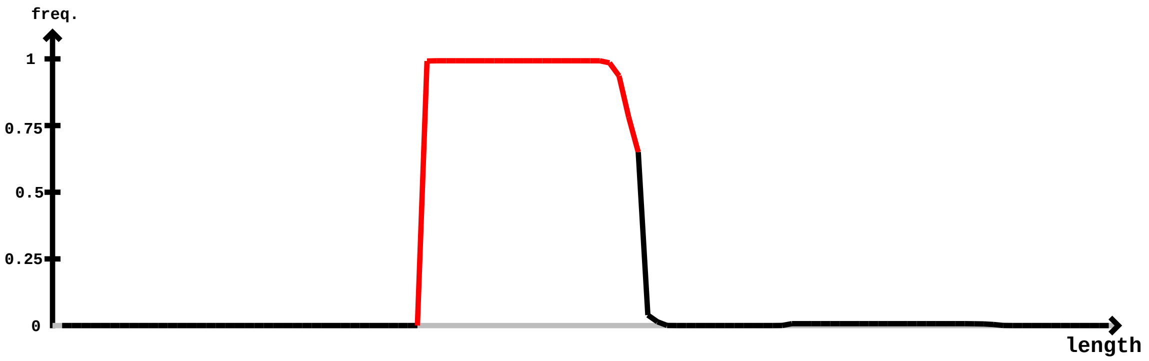

oar-miR-181a

```
5'- ugccagggcaggauccagucuucagaggacuccaaggaacauucaacgcugucggugaguuugggauuugaaaaaaccaccgaccguugacuguaccuuggguuccuua  -3'   exp
    ....(((((........)))))..(((((.(((((((.(((.((((((..(((((((.((((...........))))))))))))))))).))).))))))).))))).   reads   mm   sample
    ....................................gaacauucaacgcugucgguga...................................................   1       0    seq
    .....................................aacauucaacgcugucgg......................................................   2       0    seq
    .....................................aacauucaacgcugucggu.....................................................   122     0    seq
    .....................................aacauucaacgcugucggug....................................................   786     0    seq
    .....................................aacauucaacgcugucgguga...................................................   2469    0    seq
    .....................................aacauucaacgcugucggugag..................................................   2112    0    seq
    .....................................aacauucaacgcugucggugagu.................................................   9783    0    seq
    .....................................aacauucaacgcugucggugaguu................................................   403     0    seq
    .....................................aacauucaacgcugucggugaguuu...............................................   219     0    seq
    .....................................aacauucaacgcugucggugaguuug..............................................   6       0    seq
    ......................................acauucaacgcugucgguga...................................................   1       0    seq
    ......................................acauucaacgcugucggugagu.................................................   11      0    seq
    ......................................acauucaacgcugucggugaguuu...............................................   2       0    seq
    ..........................................................................aaccaccgaccguugacugu...............   2       0    seq
    ..........................................................................aaccaccgaccguugacugua..............   1       0    seq
    ..........................................................................aaccaccgaccguugacuguac.............   2       0    seq
    ..........................................................................aaccaccgaccguugacuguacc............   1       0    seq
    ...........................................................................accaccgaccguugacugu...............   5       0    seq
    ...........................................................................accaccgaccguugacugua..............   8       0    seq
    ...........................................................................accaccgaccguugacuguac.............   32      0    seq
    ...........................................................................accaccgaccguugacuguacc............   56      0    seq
    ...........................................................................accaccgaccguugacuguaccu...........   7       0    seq
```
